# Supplementary material for: Loss of wild-type p53 promotes mutant p53-driven metastasis through acquisition of survival and tumor-initiating properties
Source: Nat Commun. 2020 May 11;11:2333. doi: 10.1038/s41467-020-16245-1 (PMC7214469; doi:10.1038/s41467-020-16245-1)
Supplement: Supplementary file 1 — Supplementary Information [file 41467_2020_16245_MOESM1_ESM.pdf]

Supplementary information file

Loss of wild-type p53 promotes mutant p53-driven metastasis through acquisition of survival and tumor-initiating properties

Nakayama et al.

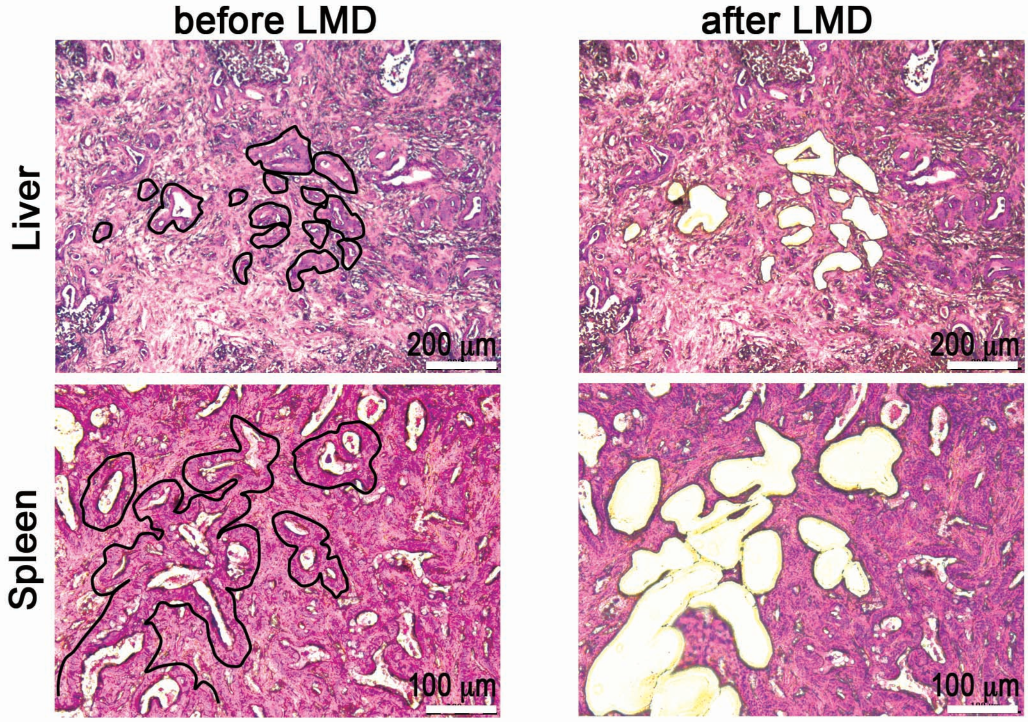

Supplementary Figure 1. Representative photographs of H&E sections before (*left*) and after dissection (*right*) by laser microdissection (LMD) of tumors from metastasized liver (*top*) and spleen (*bottom*) of NSG mice. Cutting area were circled with black lines (*left*). Bars, 200 µm (*top*) and 100 µm (*bottom*). The images are representative for three independent experiments.

Supplementary Figure 2

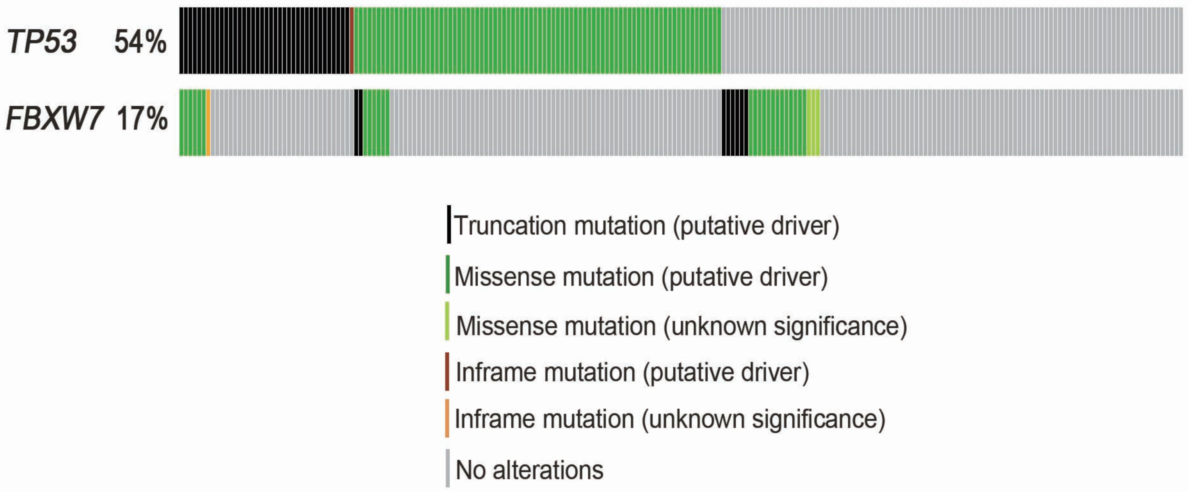

geneA	geneB	Neither	A Not B	B Not A	Both	Log Odds Ratio	p-Value	Adusted p-Value	Tendency
TP53	*FBXW7*	81	106	22	15	-0.652	0.053	0.053	Mutual exclusive

Supplementary Figure 2. The mutation profiles of *TP53* and *FBXW7* in human colorectal cancer patients from TCGA database (Nature **487**, 330-337, 2012). Mutual exclusivity was analyzed using cBioPortal (https://www.cbioportal.org). According to the data, *TP53* and *FBXW7* mutations tend to be mutual exclusive each other.

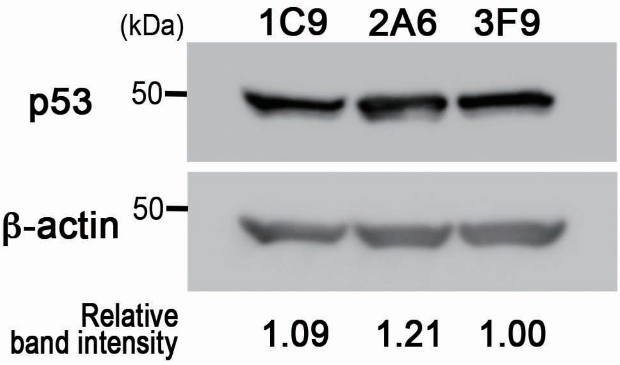

Supplementary Figure 3. Mutant p53 protein levels of 1C9, 2A6 and 3F9 AKTP$^{M/LOH}$ cells by Western blotting analysis. The ratios of p53 band intensities relative to those of β-actin are indicated. The Western blotting shown here was repeated three times with similar results, and the results of one representative experiment are shown. Source data are provided as a Source Data File.

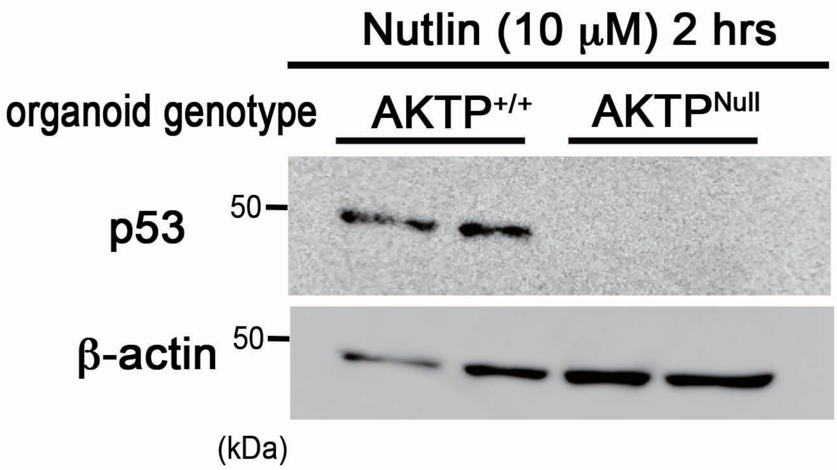

Supplementary Figure 4. *Trp53* gene knockout by CRISPR-Cas9 system was confirmed by Western blotting analysis. The organoid cells were cultured in the presence of 10 μM Nutlin which is a p53 stimulator. After 2 hrs of Nutlin stimulation, loss of p53 expression in $AKTP^{Null}$ cells was confirmed. The Western blotting shown here was repeated three times with similar results, and the results of one representative experiment are shown. Source data are provided as a Source Data File.

Supplementary Figure 5

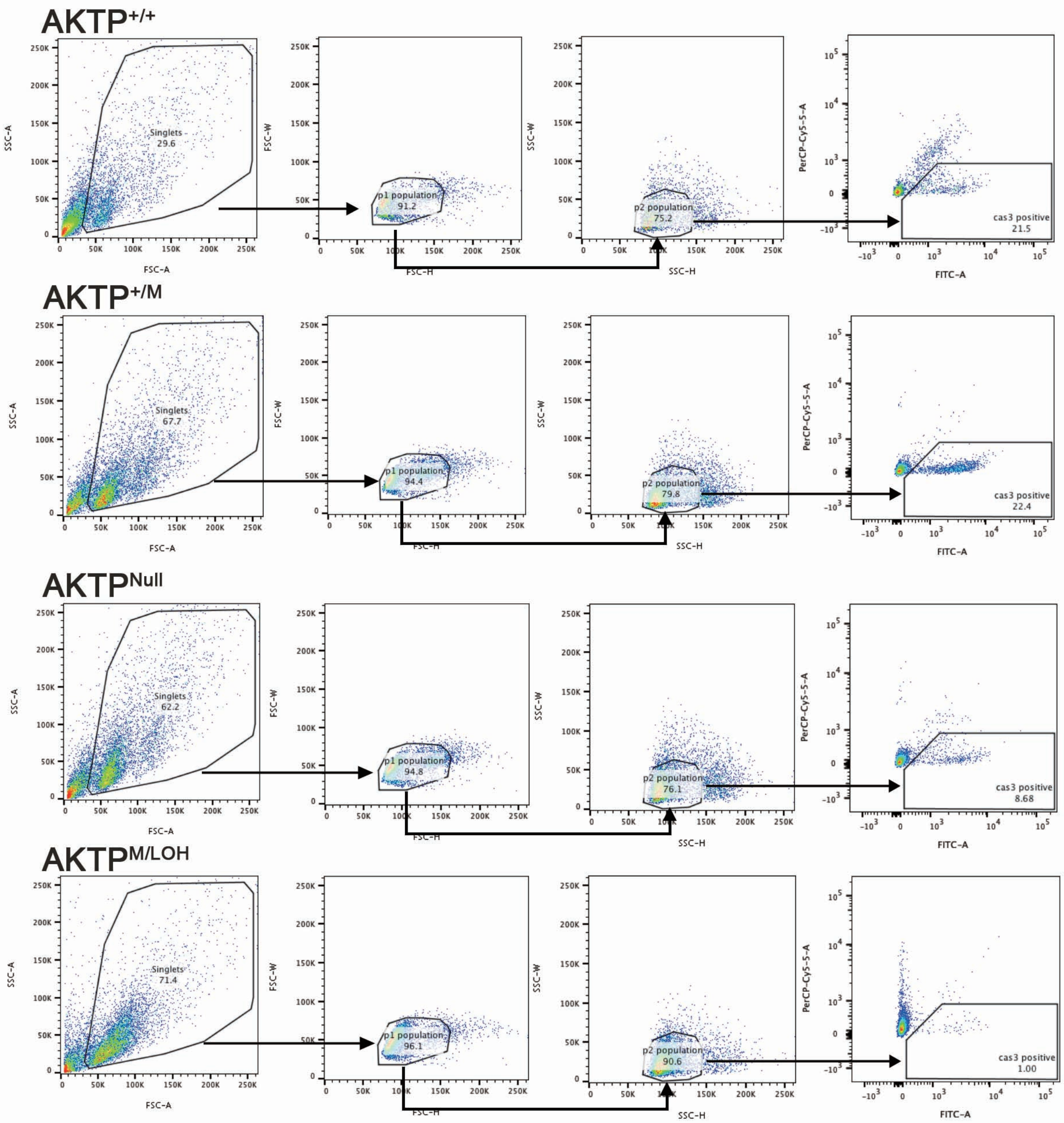

Supplementary Figure 5. Gating strategy of flow cytometry analysis for detection of cleaved Caspase 3 (Casp3) of trypsin-dissociated cells of the four different *Trp53* genotype cells ($AKTP^{+/+}$, $AKTP^{+/M}$, $AKTP^{Null}$, and $AKTP^{M/LOH}$) corresponding to Figure 4b.

Supplementary Figure 6

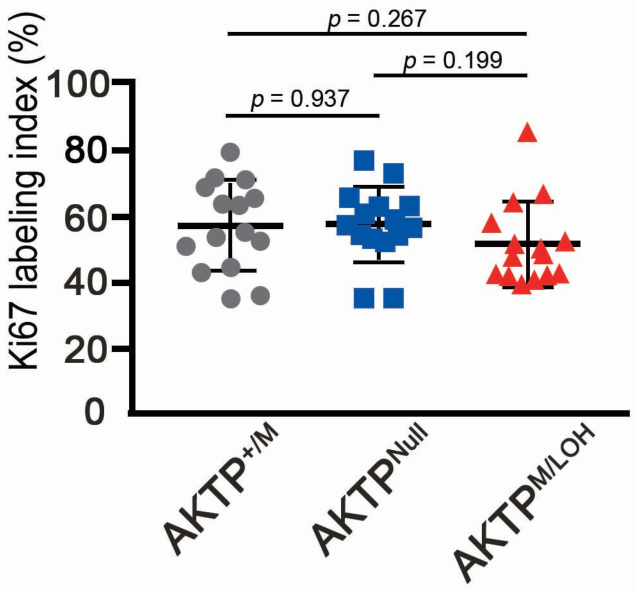

Supplementary Figure 6. The Ki67 labeling indices of the respective genotype organoids. Data are presented as mean ± s.d. with individual values, n=15 biologically independent samples, *p* values are provided in the figure. Two-sided unpaired *t*-test was used to calculate statistical difference. Source data are provided as a Source Data File.

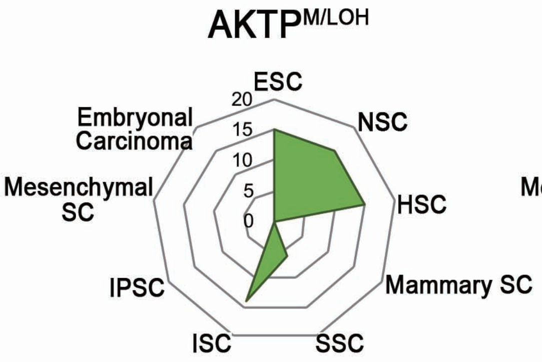

Supplementary Figure 7. The results of a StemChecker analysis using the genes that are upregulated in AKTP$^{M/LOH}$ (*left*) and AKTP^{Null} (*right*) are shown as polygon graphs.

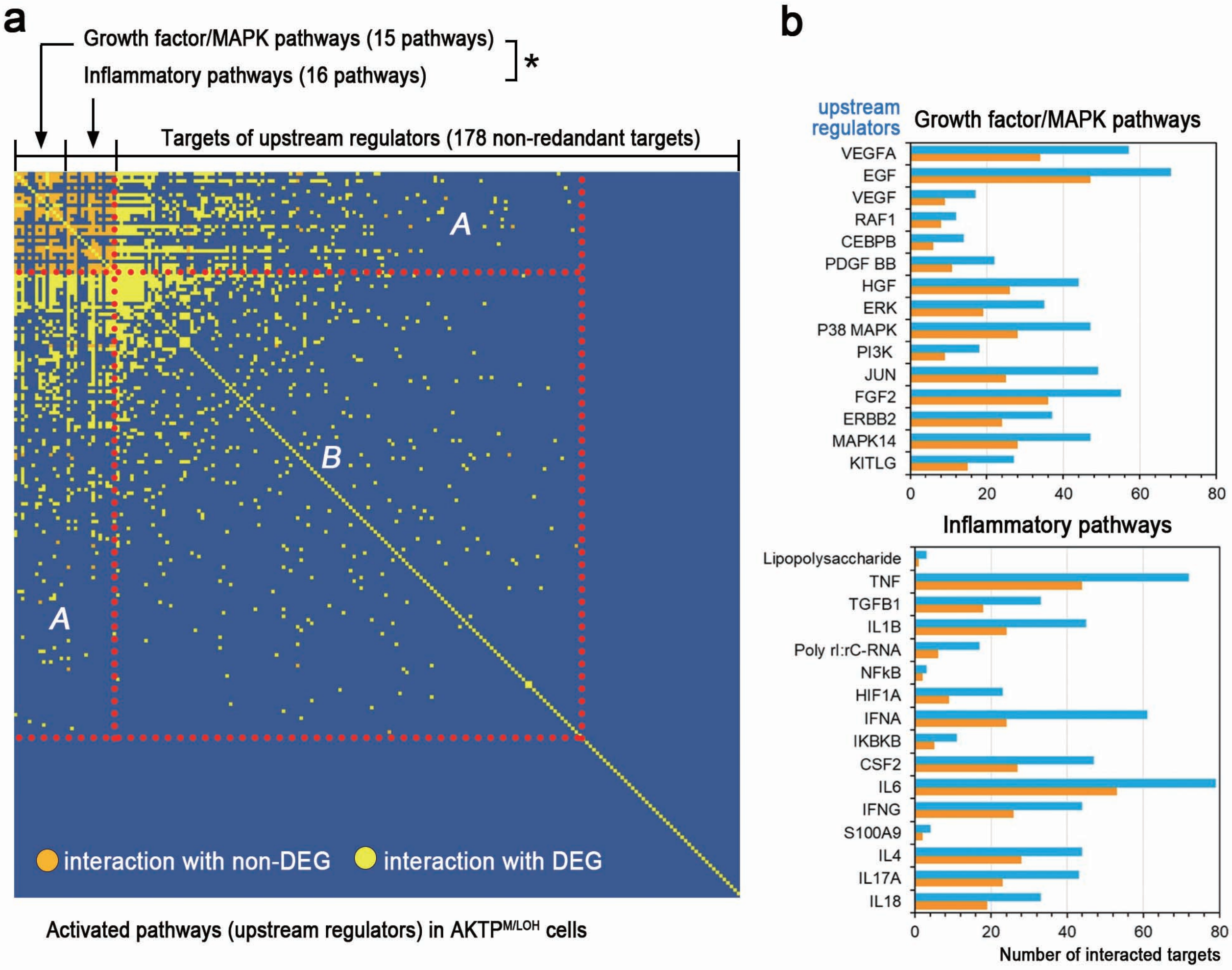

Supplementary Figure 8. Interaction of target genes of growth factor/MAPK and inflammatory pathways. (a) A heatmap showing interactions between 31 activated pathways (upstream regulators) in AKTP$^{M/LOH}$ cells and 178 their target molecules searched against STRING database with medium confidence score (> 0.4). *A*, interactions between upstream regulators and target molecules; and *B*, interactions between target molecules. (b) The exact numbers of interacted targets of each activated pathway are shown in bar graphs. *Blue bars*, interactions with all targets; and *orange bars*, interactions with differentially expressed targets.

Supplementary Figure 9

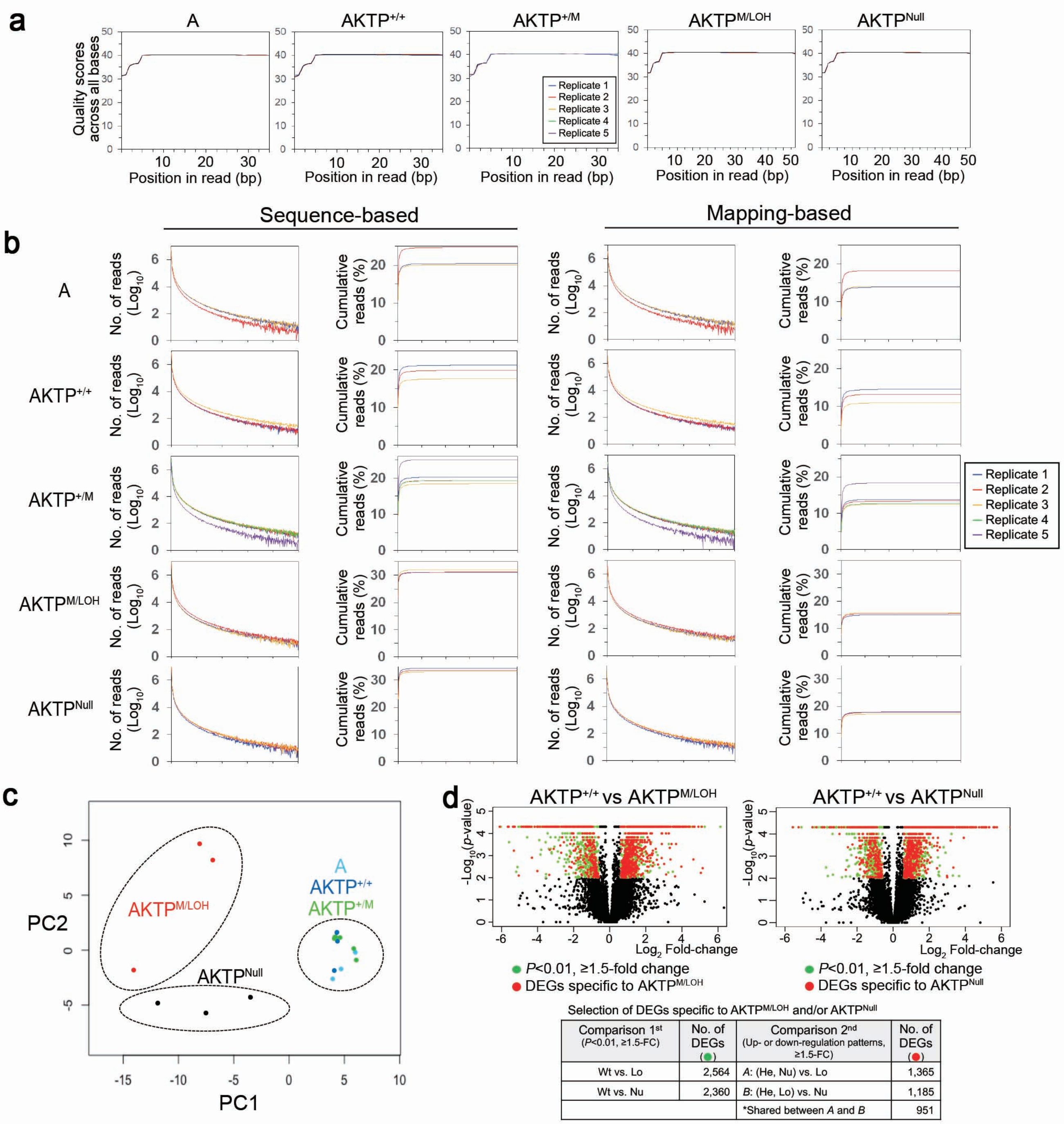

Selection of DEGs specific to $AKTP^{M/LOH}$ and/or $AKTP^{Null}$

Comparison 1st (P<0.01, ≥1.5-FC)	No. of DEGs (●)	Comparison 2nd (Up- or down-regulation patterns, ≥1.5-FC)	No. of DEGs (●)
Wt vs. Lo	2,564	*A*: (He, Nu) vs. Lo	1,365
Wt vs. Nu	2,360	*B*: (He, Lo) vs. Nu	1,185
		*Shared between *A* and *B*	951

Supplementary Figure 9. RNA-Seq quality control (QC) metrics. (a) The distribution of read quality scores. Each plot shows quality scores across all bases at each position in reads of the samples. The read quality was assessed using FastQC. In the plot, Q30 means that the base call accuracy is 99.9%. (b) Duplication rates of reads determined by sequence- (*left*) and mapping- (*right*) based approaches. In the plots, the x-axis indicates the duplication time (occurrence) and the y-axis indicates the number of uniquely mapped reads with $\log_{10}$-transformation (*left*) and the percentage of cumulative (uniquely mapped) reads, respectively. (c) PCA for the RNA-Seq samples analyzed in this study. (d) Volcano plots showing the fold-change and p value for the comparisons of $AKTP^{+/+}$ versus $AKTP^{M/LOHM}$ (*left*) and $AKTP^{+/+}$ versus $AKTP^{Null}$ (*right*). Differentially expressed genes (P < 0.01 and ≥ 1.5-fold change) are indicated with green-color dots. In addition, DEGs specific to $AKTP^{M/LOHM}$ (*left*) and/or $AKTP^{Null}$ (*right*) cells, which were filtered by comparison with other samples, are indicated with red-color dots. Of those cell-specific DEGs, 99.63% and 98.23% were identified to be less than q < 0.05, respectively (Supplementary Data 1). The selection of DEGs is indicated in the Table. Wt, $AKTP^{+/+}$; He, $AKTP^{+/M}$; Lo, $AKTP^{M/LOH}$; and Nu, $AKTP^{Null}$.

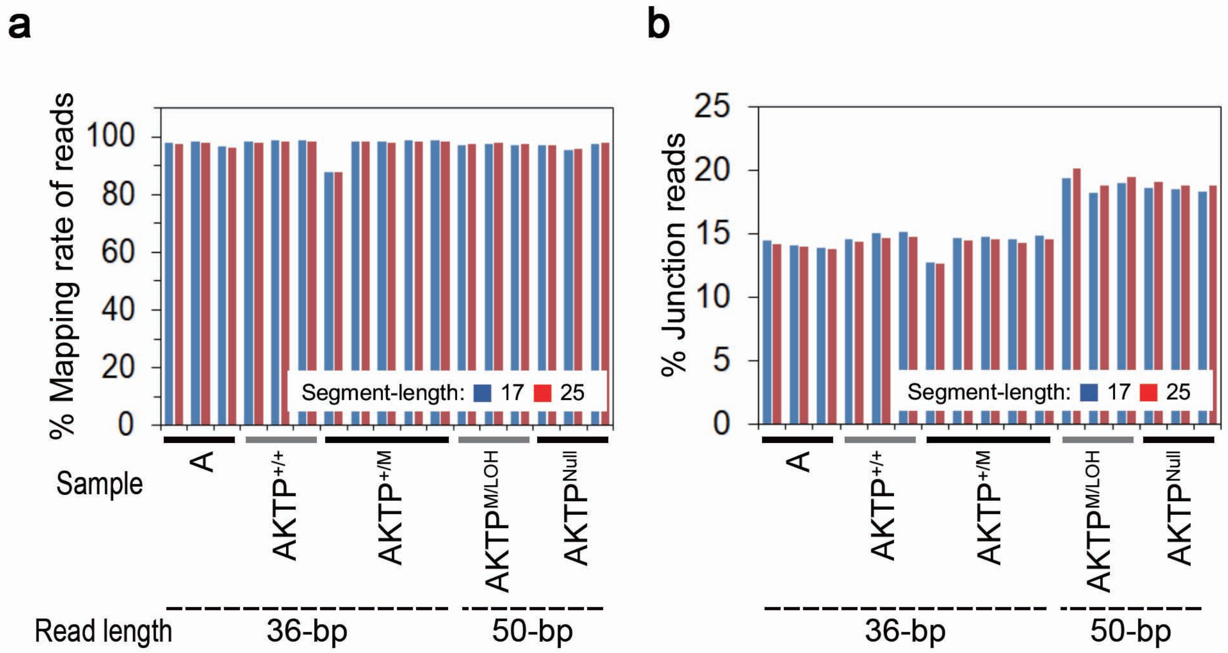

Supplementary Figure 10. The distribution of mapping reads (a) and splice junction reads (b) in RNA-Seq samples in TopHat2 alignment with segment-length 17 (*blue bar*) and 25 (*red bar*) (n=3-5 biologically independent samples).

Supplementary Table 1. Summary of RNA Sequencing data from A, AK, AKT, AKP, ATP, AKTP, and AKTPF

Sample ID (1)	Sample ID (2)	Reference	Read length (bp)	No. of read	Total length (bp)	No. of mapped read [1]	%Mapped read [2]	No. of uniquely mapped read	%Uniquely mapped read [3]
A	A1	Sakai et al.2018 [4]	36	86,054,465	3,097,960,740	83,867,032	97.46	72,062,098	85.92
	A2		36	50,684,178	1,824,630,408	49,586,957	97.84	43,103,456	86.92
	A3		36	88,721,615	3,193,978,140	85,368,428	96.22	73,187,049	85.73
AKT (or AKTP$^{+/+}$)	AKT1		36	79,990,961	2,879,674,596	78,386,978	97.99	67,534,746	86.16
	AKT2		36	80,484,273	2,897,433,828	79,187,226	98.39	68,775,854	86.85
	AKT3		36	124,732,789	4,490,380,404	122,780,583	98.43	106,039,554	86.37
AKTP$^{+/M}$	AKTP1		36	104,854,244	3,774,752,784	91,796,850	87.55	78,991,080	86.05
	AKTP2		36	94,150,995	3,389,435,820	92,478,310	98.22	79,528,289	86.00
	AKTP3		36	99,019,236	3,564,692,496	97,147,448	98.11	84,177,336	86.65
	AKTP4		36	92,796,835	3,340,686,060	91,260,484	98.34	79,047,491	86.62
	AKTP5		36	38,820,545	1,397,539,620	38,156,060	98.29	32,926,606	86.29
AKTP^{LOH}	21_1F4org	This study	50	60,488,914	3,024,445,700	58,784,894	97.18	52,784,458	89.79
	1C9		50	78,728,879	3,936,443,950	76,816,161	97.57	69,956,349	91.07
	21_1F5org		50	59,139,574	2,956,978,700	57,459,769	97.16	51,454,866	89.55
AKTP^{NULL}	AKTP_KO13		50	47,121,911	2,356,095,550	45,653,439	96.88	41,250,487	90.36
	AKTP_KO14		50	62,823,756	3,141,187,800	59,949,152	95.42	53,839,143	89.81
	AKTP_KO16		50	62,360,266	3,118,013,300	60,823,261	97.54	54,836,718	90.16

[1] Clean reads that average quality scores for all libraries are more than Q30 were mapped to the mouse reference using TopHat2.

[2] (No. of reads mapped to the mouse reference x 100) / No. of raw read

[3] (No. of uniquely mapped reads x 100) / No. of mapped read

[4] Sakai E, Nakayama M, Oshima H, Kouyama Y, Niida A, Fujii S, *et al*. Combined Mutation of Apc, Kras, and Tgfbr2 Effectively Drives Metastasis of Intestinal Cancer. Cancer Res. **2018**;78:1334-1346.
