# Supplementary material for: Loss of wild-type p53 promotes mutant p53-driven metastasis through acquisition of survival and tumor-initiating properties
Source: Nat Commun. 2020 May 11;11:2333. doi: 10.1038/s41467-020-16245-1 (PMC7214469; doi:10.1038/s41467-020-16245-1)
Supplement: Supplementary file 3 — Description of Additional Supplementary Information [file 41467_2020_16245_MOESM3_ESM.pdf]

Description of Additional Supplementary Files

File Name: Supplementary Data 1
Description: RNA sequence data of each genotype tumor organoids and the results of DEG analysis.
