# Supplementary material for: Loss of wild-type p53 promotes mutant p53-driven metastasis through acquisition of survival and tumor-initiating properties
Source: Nat Commun. 2020 May 11;11:2333. doi: 10.1038/s41467-020-16245-1 (PMC7214469; doi:10.1038/s41467-020-16245-1)
Supplement: Supplementary file 5 — Reporting Summary [file 41467_2020_16245_MOESM5_ESM.pdf]

nature research

Corresponding author(s): Masanobu Oshima

Last updated by author(s): Apr 8, 2020

Reporting Summary

Nature Research wishes to improve the reproducibility of the work that we publish. This form provides structure for consistency and transparency in reporting. For further information on Nature Research policies, see Authors & Referees and the Editorial Policy Checklist .

Statistics

For all statistical analyses, confirm that the following items are present in the figure legend, table legend, main text, or Methods section.

n/a	Confirmed	
☐	☒	The exact sample size (*n*) for each experimental group/condition, given as a discrete number and unit of measurement
☐	☒	A statement on whether measurements were taken from distinct samples or whether the same sample was measured repeatedly
☐	☒	The statistical test(s) used AND whether they are one- or two-sided<br>*Only common tests should be described solely by name; describe more complex techniques in the Methods section.*
☒	☐	A description of all covariates tested
☐	☒	A description of any assumptions or corrections, such as tests of normality and adjustment for multiple comparisons
☐	☒	A full description of the statistical parameters including central tendency (e.g. means) or other basic estimates (e.g. regression coefficient) AND variation (e.g. standard deviation) or associated estimates of uncertainty (e.g. confidence intervals)
☐	☒	For null hypothesis testing, the test statistic (e.g. *F*, *t*, *r*) with confidence intervals, effect sizes, degrees of freedom and *P* value noted<br>*Give P values as exact values whenever suitable.*
☒	☐	For Bayesian analysis, information on the choice of priors and Markov chain Monte Carlo settings
☒	☐	For hierarchical and complex designs, identification of the appropriate level for tests and full reporting of outcomes
☒	☐	Estimates of effect sizes (e.g. Cohen's *d*, Pearson's *r*), indicating how they were calculated

Our web collection on statistics for biologists contains articles on many of the points above.

Software and code

Policy information about availability of computer code

Data collection	ImageJ (https://imagej.nih.gov/nih-image/) was used to quantify signals obtained from Trp53 genomic PCR.<br>Excel (version 16.23, Microsoft) and GraphPad Prism7 were used for statistical analyses, GraphPad Prism7 was used to draw graphs.
Data analysis	Ingenuity Pathway Analysis (Ingenuity Systems: www.ingenuity.com) was used for upstream regulator analysis.<br>StemChecker (http://stemchecker.sysbiolab.eu/) was used to examine stemness signatures in gene sets.<br>cBioPortal (https://www.cbioportal.org) was used to examine mutual exclusivity of genetic mutations.<br>STRING database (https://string-db.org) has been used for interaction analysis of pathways.<br>FastQC (version 0.11.9) was used to assess the quality of RNA-seq raw data.<br>TopHat2 (version 2.1.1) was used to align RNA-seq reads to the mouse reference GRCm38.p4/mm10.<br>RSeQC (version 3.0.1) was used to determine duplication rate of reads.<br>Cufflinks (version 2.2.1) was used for gene expression quantification.<br>Cuffdiff (version 2.2.1), a part of the Cufflinks package, has been used for differential expression analysis.<br>MeV (http://mev.tm4.org) was used for hierarchical clustering analysis of expression data.

For manuscripts utilizing custom algorithms or software that are central to the research but not yet described in published literature, software must be made available to editors/reviewers. We strongly encourage code deposition in a community repository (e.g. GitHub). See the Nature Research guidelines for submitting code & software for further information.

Data

Policy information about availability of data

All manuscripts must include a data availability statement. This statement should provide the following information, where applicable:

- Accession codes, unique identifiers, or web links for publicly available datasets
- A list of figures that have associated raw data
- A description of any restrictions on data availability

The data that support the findings are available within the paper and its Supplementary Information files and from the corresponding author upon reasonable request. The source data underlying Fig. 1d-g, 2b, c, e, f, 3a, c, 4c-e, 5b-e, 6c, 7b-f and Supplementary Figs. 3, 4, 6 are provided as a Source Data file. RNA-seq data have been deposited in the DNA Data Bank Japan (DDBJ) under the accession numbers: #DRA005647 and #DRA008701 and all data sets have been released publicly. cBioPortal (https://www.cbioportal.org) has been used to examine mutual exclusivity of genetic alterations in human CRC. STRING database (https://string-db.org) has been used for interaction analysis of pathways.

Field-specific reporting

Please select the one below that is the best fit for your research. If you are not sure, read the appropriate sections before making your selection.

[x] Life sciences [ ] Behavioural & social sciences [ ] Ecological, evolutionary & environmental sciences

For a reference copy of the document with all sections, see nature.com/documents/nr-reporting-summary-flat.pdf

Life sciences study design

All studies must disclose on these points even when the disclosure is negative.

Sample size	No statistical methods were used to determine the sample size. Required sample sizes were determined based on previous experiments performed in our laboratory (Oncogene 2017; 36, 5885-5896; Cancer Res 2018; 78, 1334-1346; FASEB J 2019; 33, 1873-1886).
Data exclusions	No data was excluded from the analysis.
Replication	Experiments were performed at least three times independently. For most experiments, the mean and standard deviation or the individual results are shown. For western blots, genomic PCR, immunohistochemistry and H&E staining, representative results are shown. All attempts at replication were successful.
Randomization	Mice were randomly selected after housing in SPF for 1 week after purchase, and were transplanted with organoids or organoid-derived cells.
Blinding	Investigators were blinded during data collection and analysis.

Reporting for specific materials, systems and methods

We require information from authors about some types of materials, experimental systems and methods used in many studies. Here, indicate whether each material, system or method listed is relevant to your study. If you are not sure if a list item applies to your research, read the appropriate section before selecting a response.

Materials & experimental systems

n/a	Involved in the study	
[ ]	[x]	Antibodies
[ ]	[x]	Eukaryotic cell lines
[x]	[ ]	Palaeontology
[ ]	[x]	Animals and other organisms
[x]	[ ]	Human research participants
[x]	[ ]	Clinical data

Methods

n/a	Involved in the study	
[x]	[ ]	ChIP-seq
[ ]	[x]	Flow cytometry
[x]	[ ]	MRI-based neuroimaging

Antibodies

Antibodies used	Anti αSMA (Mouse monoclonal 1A4, #A2547-100UL), Sigma. Anti E-cadherin (Goat polyclonal, #AF748), R&D. Anti p53 (CM5) (Rabbit polyclonal, #P53-CM5P), Leica Biosystems. Anti p53 (Mouse monoclonal 1C12, #2524T), Cell Signaling. Anti Ki67 (Rabbit monoclonal, #ab16667), Abcam. Anti Sox17 (Goat polyclonal, #AF1924), R&D. Anti beta Actin (Mouse monoclonal, #A5441), SIGMA. Anti cleaved caspase 3 (Asp175) (Alexa Fluor® 488 conjugate, #9669S), Cell Signaling. Alexa Fluor® 594- and Alexa Fluor® 488-conjugated antibodies, Molecular Probes.
Validation	Anti αSMA (Sigma) https://www.sigmaaldrich.com/catalog/product/sigma/a2547?lang=ja&region=US<br>Anti E-cadherin (R&D) https://www.rndsystems.com/products/human-mouse-e-cadherin-antibody_af748

Anti p53 (CM5) (Leica Biosystems) https://shop.leicabiosystems.com/us/ihc-ish/ihc-primary-antibodies/pid-p53-protein-cm5
Anti p53 (1C12) (Cell Signaling) https://en.cellsignal.jp/products/primary-antibodies/p53-1c12-mouse-mab/2524
Anti Ki67 (Abcam) https://www.abcam.co.jp/ki67-antibody-sp6-ab16667.html
Anti Sox17 (R&D) https://www.rndsystems.com/search?keywords=Sox17+antibody+AF1924
Anti beta Actin (SIGMA) https://www.sigmaaldrich.com/catalog/product/sigma/a5441?lang=en&region=CA
Anti cleaved caspase 3 (Asp175) (Cell Signaling) https://en.cellsignal.jp/products/antibody-conjugates/cleaved-caspase-3-asp175-antibody-alexa-fluor-488-conjugate/9669
Alexa Fluorے 594- and Alexa Fluorے 488-conjugated antibodies (Molecular Probes) https://www.thermofisher.com/jp/ja/home/brands/molecular-probes/key-molecular-probes-products/alexa-fluor/alexa-fluor-products.html

Eukaryotic cell lines

Policy information about cell lines

Cell line source(s)	CMT93 cells were purchased from ATCC (cat# EC89111413). We established AKTP+/M, ATP+/M, AKP+/M, AKF and AKTFP+/M organoid cell lines from mouse intestinal tumors carrying mutations in the combinations of Apc Kras Tgfbr2 Trp53+/R270H, Apc Tgfbr2 Trp53+/R270H, Apc Kras Trp53+/R270H, Apc Kras Fbxw7, and Apc Kras Tgfbr2 Fbxw7 Trp53+/R270H, respectively, and used in this study. These cell lines can be shared upon reasonable request.
Authentication	CMT 93 cells were authenticated by ATCC. All organoids and derived cell lines were authenticated by genotyping PCR.
Mycoplasma contamination	All organoid lines and cell lines (involving CMT93 cells) were tested negative for mycoplasma contamination.
Commonly misidentified lines (See ICLAC register)	None.

Animals and other organisms

Policy information about studies involving animals; ARRIVE guidelines recommended for reporting animal research

Laboratory animals	Female NSG mice between 6 and 7 weeks of age were used for this study. The mice were housed in a 12-h light:dark cycle at 23°C±2°C temperature with relative humidity of 50±20 %, and given ad-libitum access to food and water for the duration of the study. Mice were housed in specific-pathogen-free (SPF) conditions and cared for in accordance with Fundamental Guidelines for Proper Conduct of Animal Experiment and Related Activities in Academic Research Institutions under the jurisdiction of the Ministry of Education, Culture, Sports, Science and Technology of Japan.
Wild animals	This study did not use wild animals.
Field-collected samples	This study did not use samples collected from the fields.
Ethics oversight	All mouse experiments were carried out according to the protocol approved by the Committee on Animal Experimentation of Kanazawa University.

Note that full information on the approval of the study protocol must also be provided in the manuscript.

Flow Cytometry

Plots

Confirm that:

- [x] The axis labels state the marker and fluorochrome used (e.g. CD4-FITC).
- [x] The axis scales are clearly visible. Include numbers along axes only for bottom left plot of group (a 'group' is an analysis of identical markers).
- [x] All plots are contour plots with outliers or pseudocolor plots.
- [x] A numerical value for number of cells or percentage (with statistics) is provided.

Methodology

Sample preparation	AKTP cell lines were established from the AKTP organoids as described in the Methods.
Instrument	FACSCanto II (BD Biosciences)
Software	FACSDiva software (BD) was used for data collection and Flowjo v10 for data analysis.
Cell population abundance	One-two hundred thousand dissociated-single cells isolated from organoids per genotype were fixed and stained for the cleaved caspase-3 antibody, analized by FACS.
Gating strategy	Debris was removed from the main cell population using the FSC/SSC gating. Positively population for each samples was defined on the basis of unstained cells. Identical positively population was applied to all genotype samples. Representative plots are

provided with Figure 4b.

- [x] Tick this box to confirm that a figure exemplifying the gating strategy is provided in the Supplementary Information.
